# Spin wavepackets in the Kagome ferromagnet Fe_3_Sn_2_: Propagation and precursors

**DOI:** 10.1073/pnas.2220589120

**Published:** 2023-05-15

**Authors:** Changmin Lee, Yue Sun, Linda Ye, Sumedh Rathi, Kevin Wang, Yuan-Ming Lu, Joel Moore, Joseph G. Checkelsky, Joseph Orenstein

**Affiliations:** ^a^Materials Science Division, Lawrence Berkeley National Laboratory, Berkeley, CA 94720; ^b^Department of Physics, Hanyang University, Seoul 04763, Republic of Korea; ^c^Department of Physics, University of California, Berkeley, CA 94720; ^d^Department of Physics, Massachusetts Institute of Technology, Cambridge, MA 02139; ^e^Department of Physics, The Ohio State University, Columbus, OH 43210

**Keywords:** spin transport, time-resolved Kerr micrsocopy, magnetostatic spin waves

## Abstract

The propagation of spin waves in magnetically ordered systems has emerged as a potential means to shuttle quantum information over large distances. Conventionally, the arrival time of a spin wavepacket at a distance, *d*, is assumed to be determined by its group velocity, *v*_*g*_. Here, we report time-resolved optical measurements of wavepacket propagation in the Kagome ferromagnet Fe_3_Sn_2_ that demonstrate the arrival of spin information at times significantly less than *d*/*v*_*g*_. We show that this spin wave “precursor” originates from the interaction of light with the unusual spectrum of magnetostatic modes in Fe_3_Sn_2_. Related effects may have far-reaching consequences toward realizing long-range, ultrafast spin wave transport in both ferromagnetic and antiferromagnetic systems.

## Significance

Wavepackets of magnetization in magnetically ordered materials have emerged as a potential means to shuttle quantum information over large distances. A particularly promising platform is quasi-two-dimensional magnets in which the spins within each atomic plane are parallel but interplane order can be ferromagnetic or antiferromagnetic. In this work, we use ultrashort light pulses to generate spin wavepackets in a kagome-layered ferromagnet and to follow their subsequent motion. A significant result is that the arrival of magnetization at a remote location occurs in a time far shorter than expected from the spin wave velocity. We show that this “precursor” originates from the long-range magnetic dipole interaction. Related effects may have far-reaching consequences toward realizing long-range transport of spin information.

Harnessing electron spin is one of the central goals of condensed matter physics. A particularly exciting direction is the coupling of spin to charge and lattice degrees of freedom to provide interconnections in hybrid quantum systems. To this end, it is essential to understand and control the generation, propagation, and detection of spin information. Recent progress in magnetically ordered systems has shown promise in using propagating spin waves—collective excitations of the electron spins—to transport information over large distances (1–5). Increasingly, attention has focused on quasi-two-dimensional (2D) layered magnets in which spins within each plane are parallel but the interplane order can be ferromagnetic (6), antiferromagnetic (7), or even helical (8).

An important subset of such systems is “easy-plane” magnets in which spins are oriented parallel to the planes but without a preferred direction within the plane. As a result of the symmetry with respect to in-plane spin rotation, the out-of-plane, or *z* component of the magnetization, *M*_*z*_, is a conserved quantity. Theoretically, *M*_*z*_ can exhibit ballistic, diffusive, hydrodynamic, or even superfluid regimes of transport (9). However, in real 2D easy-plane magnets, this rotational symmetry is broken, although weakly, by the anisotropy of the underlying lattice. This fact has driven theoretical studies of the consequences of rotational symmetry breaking and approaches to mitigating its effects (10, 11).

At low temperatures, Fe_3_Sn_2_ exemplifies an easy-plane system of the class introduced above, in which the spins experience weak anisotropy resulting from the discrete 3-fold rotational symmetry of the rhombohedrally stacked Kagome lattice (12–14). In this work, we study the propagation of spin wavepackets in Fe_3_Sn_2_, using temporal and spatially resolved optical techniques to probe their amplitude, frequency, and velocity. In our pump/probe measurement scheme, the pump pulse excites a spin wavepacket whose propagation is detected by a time-delayed and spatially separated probe pulse through the magneto-optic Kerr effect (MOKE) (15, 16) or optical birefringence (17). The range of wavevectors that comprise the spin wavepacket is determined by the Fourier transform of the real space excitation density, which is typically Gaussian.

Since the size of the focused laser spot is diffraction limited, the excited wavevectors are typically within the range of inverse micrometers (μm^−1^). In this long wavelength regime, the propagation of spin is dominated by magnetic dipole interactions, drastically altering the properties that arise from short-ranged exchange interactions alone (18, 19). Excitations in this regime are referred to as magnetostatic spin waves (MSWs) although they are fully dynamic; the term arises because their dispersion relations can be obtained within the magnetostatic approximation, ∇ × **H** = 0, which is valid because spin wave velocities are much smaller than the speed of light.

Given the long-range nature of the dipole interaction, MSWs are particularly sensitive to both the shape of the medium and magnetic anisotropy. Damon and Eshbach (DE) (18) obtained MSW dispersion relations for a magnetic slab with uniaxial anisotropy, associated with either an easy axis or applied magnetic field. However, as we demonstrate below, spin wavepacket propagation in Fe_3_Sn_2_ shows novel properties that cannot be described by the DE relations, including the remarkable observation that spin excitations can be detected remotely at a time much shorter than would be inferred from the spin wave velocity. In the theoretical component of our study, we use both analytical calculations and numerical modeling to show that these effects are accounted for by extending the DE formalism to the easy-plane systems of current interest. Although the theory presented below assumes ferromagnetic order between the planes, as in Fe_3_Sn_2_, it applies to antiferromagnetic order as well, for example, the antiferromagnetic version of the theory (20), a quantitative explanation for the recently discovered surprising properties of spin wavepacket propagation in the 2D van der Waals antiferromagnet CrSBr (21).

## Results

### Magnetic Field Dependence of Spin Wave Frequency.

**Fig. 1.**
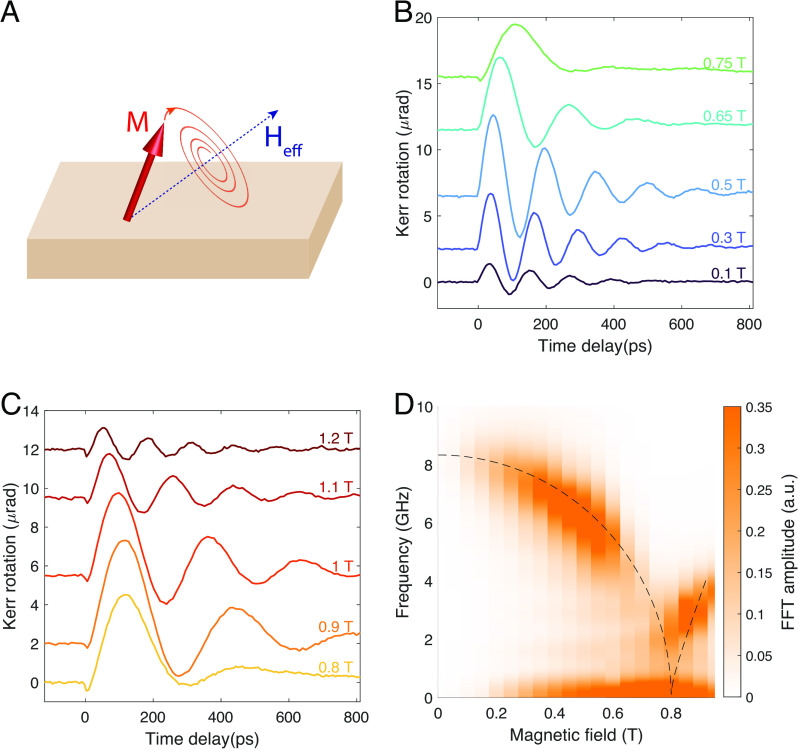
(*A*) Illustration of magnetization spiraling to align with *H*_eff_. (*B* and *C*) Kerr rotation as a function of pump–probe delay shown for applied magnetic fields ≤0.75 and > 0.75 T, respectively. The curves are offset for clarity. (*D*) Amplitude of Fourier transforms of the time series plotted in (*B*) and (*C*) shown in the frequency-field plane. The dashed line indicates the fit to Eq. **1**. All data were taken at *T* = 2.5 K.

Prior to measurements of spin transport, the anisotropy parameters of Fe_3_Sn_2_ were determined using the time-resolved magneto-optic Kerr effect (TR-MOKE). In this method, spin waves are generated and detected by pulses of light. The amplitude of photoexcited spin waves was found to be independent of polarization, indicating that they are generated by an incoherent mechanism, in which the pump pulse generates a nonequilibrium electron distribution and partial demagnetization. Demagnetization, in turn, leads to a change in the direction of the effective anisotropy field and therefore misalignment between the magnetization, **M**, and the effective anisotropy field, **H**_eff_. The resulting torque causes **M** to precess, as illustrated in Fig. 1*A*. The precession leads to oscillations of the component of magnetization parallel to the optic axis, *M*_*z*_, which are detected via the polar Kerr effect (15, 16, 22).

Fig. 1 *B* and *C* show oscillations of *M*_*z*_ as detected by the TR-MOKE for several magnetic fields applied in the *z* direction. Fig. 1*D* displays the Fourier transform of the oscillations in the frequency-magnetic field plane; the dashed line is a fit to a model described below. This dependence of spin wave (SW) frequency on the field is characteristic of a ferromagnet whose biaxial anisotropy can be described by the free energy $F_{A}=\left(-K_{x}M_{x}^{2}+K_{z}M_{z}^{2}\right)/M_{s}^{2}$, where *K*_*x*_ and *K*_*z*_ are the in- and out-of-plane anisotropy energies (*K*_*x*_, *K*_*z*_ > 0) and *x* is a preferred magnetization direction within the plane. The origin of the in-plane anisotropy is discussed in *SI Appendix*, sections I and II, which present a microscopic model whose low-temperature, broken-symmetry phase is described by *F*_*A*_ for small fluctuations of the ferromagnetic order parameter. The theoretically predicted dependence of SW frequency on magnetic field, *ν*(*H*_*z*_), for a biaxial ferromagnet is (23, 24)

[1] $$\begin{array}{c} \nu \left(H_{z}\right)=\left\{\begin{array}{cc} \frac{\gamma \sqrt{\left(H_{s}^{2}-H_{z}^{2}\right)K_{x}\left(K_{x}+K_{z}+2\pi M_{s}^{2}\right)}}{2\pi (K_{x}+K_{z})} & 0⩽H_{z}⩽H_{s}\\ \frac{\gamma }{2\pi }\sqrt{\left(H_{z}-\frac{2K_{z}}{M_{s}}\right)\left(H_{z}-H_{s}\right)} & H_{z}>H_{s}, \end{array}\right. \end{array}$$

where *H*_*s*_ is the saturation field along the *z* direction, *M*_*s*_ is the saturation magnetization, and *γ* is the gyromagnetic ratio. Eq. **1** accurately describes the full field dependence of the TR-MOKE frequencies observed in our experiment (for a derivation, *SI Appendix*, section III). The fit (dashed line in Fig. 1*D*) yields anisotropy parameters *K*_*x*_ ≈ 1.76 × 10^4^ J/m^3^ and *K*_*z*_ ≈ 2.26 × 10^5^ J/m^3^, consistent with weak anisotropy within an easy plane, and *γ* = 1.7 × 10^11^*T*^−1^*s*^−1^. The saturation magnetization value, *M*_*s*_ = 6.5 × 10^5^J/m^3^*T*^−1^, was determined from magnetization vs. field measurements (13). Note that while the theoretical prediction (Eq. **1**) is written in Gaussian (cgs) units, we have followed the convention of expressing magnetic properties in the SI system.

### Detection of Spin Propagation Using Scanning TR-MOKE Microscopy.

**Fig. 2.**
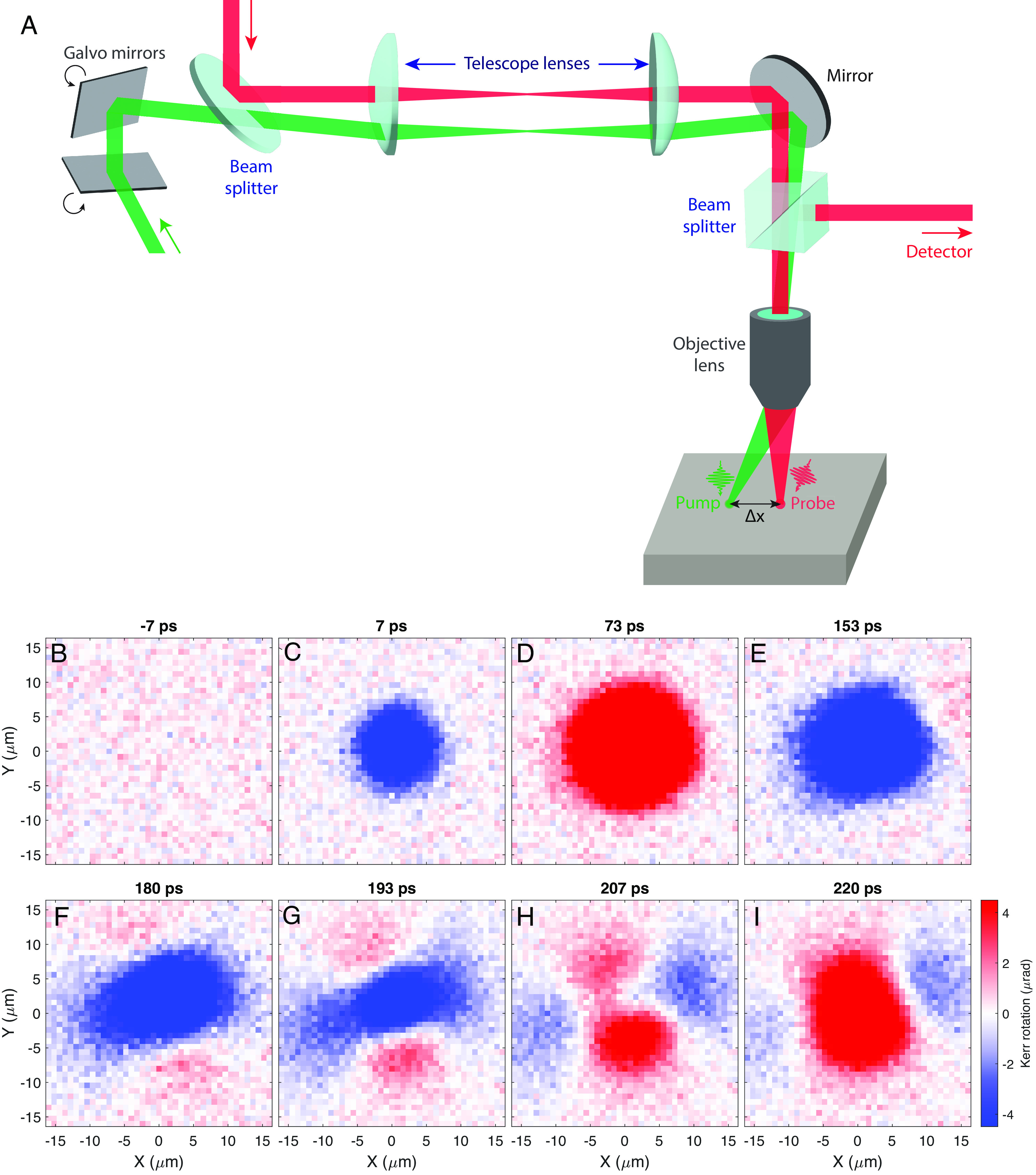
Time-resolved MOKE microscopy (*A*) Overview of the experimental setup. The 2D galvo mirrors and the 4*f* optical geometry enable scanning of the pump laser beam. (*B*–*I*) Snapshots of 2D MOKE maps. The images are obtained by rastering the pump beam and sampling the Kerr signal at fixed time delay between pump and probe pulses. For time delay *t* ≥ 180 ps (*F*–*I*), the propagation is clearly anisotropic, with contrasting properties along two principal axis directions. All measurements were performed at *T* = 2.5 K with an out-of-plane field of 0.5 T.

We now describe extending time-resolved measurements to the spatial domain. A simplified layout of the setup for TR-MOKE microscopy is shown in Fig. 2*A*. The 4*f* optical system equipped with 2-axis galvo-driven mirrors enables continuous scanning of the pump focus in two dimensions while the location of the probe is fixed (25). Spin waves photoexcited in one location can be probed remotely at a subsequent time, enabling an all-optical ultrafast investigation of SW transport with micron-scale spatial resolution and submicroradian polarization sensitivity.

Fig. 2*B*–*I* show TR-MOKE maps measured at various pump–probe time delays (*Δt*) ranging from −7 to +220 ps. Here, the *x* and *y* axes refer to the separation between the pump and probe beams. Shortly after photoexcitation (*Δt* < 100 ps), the transient changes in magnetization are isotropic (Fig. 2*C*–*E*). However, at *Δt* = 180 ps, (Fig. 2*F*) clear evidence of anisotropic propagation is observed, with a stronger MOKE signal along ∼ < *SPSDOUBLEDOLLAR* > 20 ° /200° with respect to the *x* axis. At longer times (Fig. 2*G* –*I*), the contrasting nature of propagation between the 20 ° /200° and 110 ° /290° becomes increasingly clear (angular dependence of the anisotropic propagation is further discussed in *SI Appendix*, section IV).

**Fig. 3.**
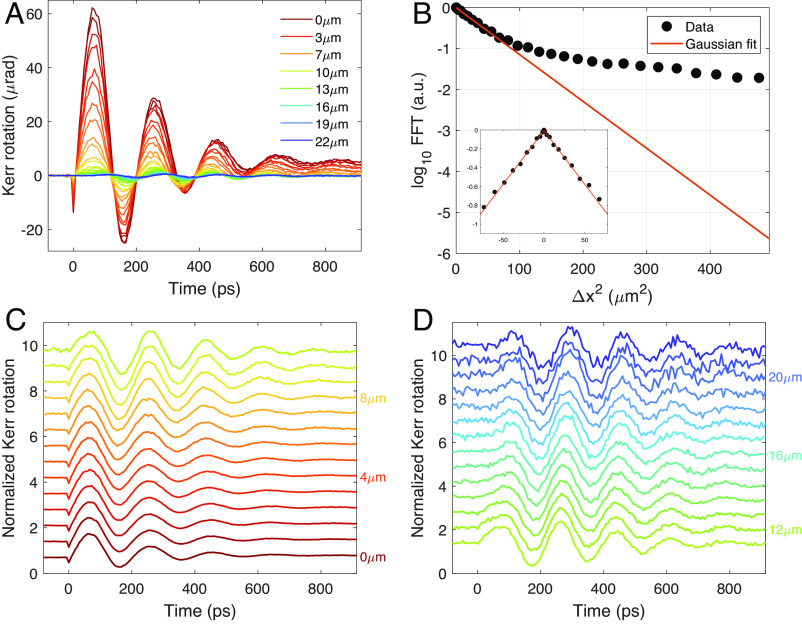
(*A*) TR-MOKE traces at different values of spatial separation (*Δx*) between the pump and probe beams. (*B*) The log of the amplitude of the Fourier transform of the data shown in (*A*) is plotted vs. (*Δx*)^2^ (black dots). The red line is the rate of decrease expected in the absence of propagation. (*C* and *D*) Normalized TR-MOKE traces at separations for *Δx* < 10 μm and *Δx* > 10 μm, respectively, illustrating the change in the envelope function from exponential to Gaussian. All measurements were taken at *T* = 2.5 K under an out-of-plane field of 0.5 T.

To further characterize spin propagation, we consider the rate of decay of the TR-MOKE oscillations with increasing propagation distance. In Fig. 3*A*, we plot TR-MOKE time traces for several values of pump–probe separation along the major propagation axis (20 ° /200 °). The amplitude at a given separation is determined from the peak value of the Fourier transform of the oscillations. The log of this amplitude is plotted as a function of (*Δx*)^2^ as solid circles in Fig. 3*B*. For small separations, the amplitude decreases in proportion to *e*^−(*Δx*/*σ*)^2^^, where *σ* ∼ 6 μm. In this regime, the decay of the amplitude reflects the spatial overlap of pump and probe foci (with full width at half-maximum (FWHM) spot sizes of 6 and 5 μm, respectively) as would be expected in the absence of propagation. However, for larger *Δx*, spin propagation becomes evident; for *Δx* > 10 μm, the TR-MOKE amplitude deviates from a Gaussian and at *Δx* = 20 μm is four orders of magnitude larger than can be accounted for by spatial overlap.

The distinction between the overlap and propagation regimes is also seen by normalizing the TR-MOKE traces to the amplitude at zero separation. Fig. 3*C* shows the normalized signals for *Δx* < 10 μm, which is in the Gaussian regime of Fig. 3*B*. For these separations, the envelope of the TR-MOKE oscillations decays monotonically with increasing time, consistent with a simple damped response. However, at separations greater than 10 μm, shown in Fig. 3*D*, the envelope peaks at a nonzero time delay, as expected for a propagating wavepacket. Focusing on the arrival time of the wavepacket at the largest measured separation of 22 μm reveals another surprising feature. Notice that the first clear indication that spin waves have reached this distance occurs at ≈100 ps, from which we estimate an effective velocity of ≈2 ×10^7^ cm/s. This velocity is six orders of magnitude larger than the group velocity inferred from neutron scattering measurements (26). In the following section, we show that discrepancy is resolved by considering wavepacket propagation in the magnetostatic regime.

## Discussion

As mentioned in the introduction, spin wavepacket propagation in Fe_3_Sn_2_ cannot be described by the DE dispersion relations for either surface or bulk modes. For example, the DE surface mode is nonreciprocal, with a single direction of propagation that is reversed for the two opposing surfaces. Instead, we observe reciprocal propagation that is symmetric with respect to wavevector **k** → −**k**. The volume modes, although reciprocal, propagate only along one axis, whereas we observe propagating modes along two principal axes in the plane. Furthermore, the bidirectional DE volume mode is “backward moving” in the sense that its phase and group velocities are opposite, whereas we find that the two principal axes of propagation exhibit forward and backward modes, respectively. As we show below, extending the DE calculation to nearly easy-plane systems accounts for the features observed in our spin transport measurements.

### Magnetostatic Spin Waves under Biaxial Anisotropy.

**Fig. 4.**
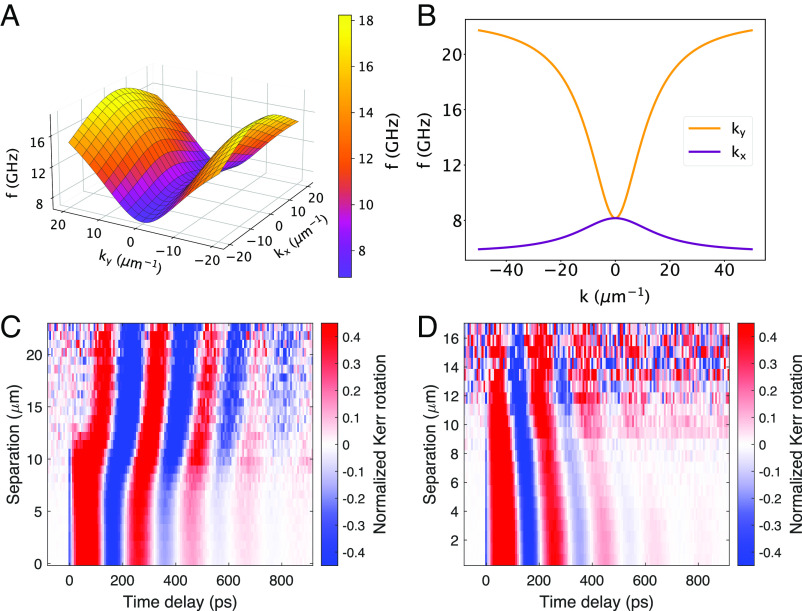
Magnetostatic waves (MSWs) under biaxial anisotropy. (*A*) Three dimensional representation of the calculated MSW dispersion of Fe_3_Sn_2_ as a function of *k*_*x*_ and *k*_*y*_ evaluated at *k*_*z*_ = 1 μm^−1^. A saddle point can be observed at the origin. (*B*) Frequency-momentum cuts at *k*_*x*_ = 0 (purple) and *k*_*y*_ = 0 (orange) illustrating forward propagation along *k*_*y*_ (purple) and backward propagation along *k*_*x*_ (orange). (*C* and *D*) Plots of the TR-MOKE amplitude in the *Δx*, *t* plane measured along the two principal axes of propagation show forward and backward propagation, respectively.

We consider a geometry with the equilibrium magnetization in the plane and parallel to one of the easy axes (27, 28). Maxwell’s equations in the magnetostatic regime, ∇ ⋅ **B** = ∇ × **H** = 0, together with the Landau–Lifshitz equation,

[2] $$\begin{array}{c} \frac{\partial M}{\partial t}=-\gamma M\times H_{\mathrm{eff}}, \end{array}$$

where $H_{\mathrm{eff}}$ is the sum of the anisotropy field and the dynamical field h, form a closed set that yields the normal modes of magnetization in the long-wavelength regime. To illustrate the resulting MSW dispersion, Fig. 4*A* shows the calculated spin wave frequency in the *k*_*x*_, *k*_*y*_ plane for fixed *k*_*z*_=1 μm^−1^. Line cuts through this plane defined by *k*_*x*_ = 0 (purple) and *k*_*y*_ = 0 (orange) plotted in Fig. 4*B* show forward propagation along the *y* direction and backward along *x*, with a saddle point at **q** = 0. When *K*_*z*_ > *K*_*x*_, as in Fe_3_Sn_2_, the velocity is larger along *k*_*y*_. This dispersion relation was also reproduced through micromagnetic simulations. These predictions are unique to biaxial ferromagnets and clearly distinct from the uniaxial (DE) limit, in which there are no forward-propagating reciprocal modes (*SI Appendix*, sections V and VI for the calculations and numerical simulation of the MSW dispersion relations).

The prediction of a saddle dispersion relation was tested by measuring the TR-MOKE oscillations as a function of pump/probe separation along the two principal axes of propagation identified in the maps shown in Fig. 2. The results are presented in Fig. 4 *C* and *D* as color plots in the time-separation plane. The slope of the lines of the constant phase distinguishes forward- vs. backward-propagating modes. In agreement with our theoretical prediction for the biaxial ferromagnet, modes with wavevector perpendicular to M are forward-propagating and backward-propagating for wavevectors parallel to M.

### Spin Wavepacket Propagation.

**Fig. 5.**
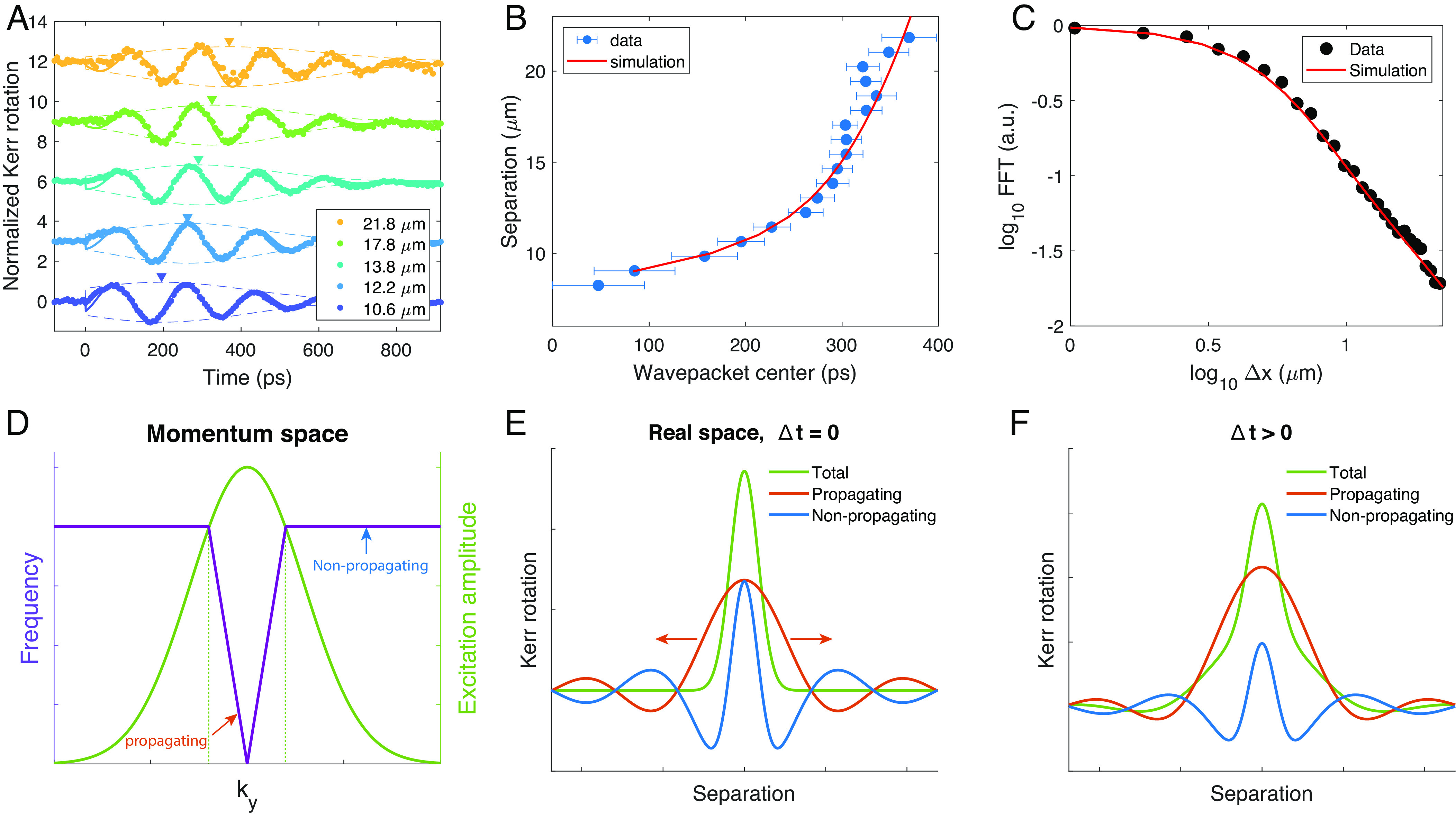
(*A*) Normalized TR-MOKE amplitude *vs.t* for values of *Δx* > 10 μm with arrowheads indicating the center of the wavepacket. (*B*) The solid circles show the wavepacket center as a function of time. The red line is a fit based on the calculated MSW dispersion relation. (*C*) A double logarithmic plot of the amplitude of the wavepacket vs. pump–probe separation (solid circles) and the fit (red line) using the same parameters as in (*B*). (*D*–*F*) Illustration of the physical origin of the wavepacket precursor. (*D*) Shown as a green line is the Gaussian distribution of wavevectors excited by the pump beam. The purple line is an approximation to the MSW dispersion for a value of *k*_*z*_ that is within the range of excited in-plane wavevectors. The regimes with group velocity *v*_*g*_ > 0 and *v*_*g*_ = 0 are indicated. (*E*) Red and blue lines show the contributions to the total Kerr rotation from the propagating and nonpropagating modes, respectively, evaluated at *t* = 0. As expected, their sum yields a Gaussian profile corresponding to the initial photoexcited state. (*F*) As the profile of the propagating modes evolves for *t* > 0, the oscillations associated with the nonpropagating modes no longer cancel. The total Kerr rotation shown in green reveals evidence of propagation even at separations greater than *v*_*g*_*t*.

We turn next to the dynamics of wavepackets whose motion is determined by the dispersion relation illustrated in Fig. 4. The primary goal is to understand how spin information propagates in the MSW regime. Fig. 5*A*–*C* show the comparison of experiment and theory for the amplitude and position of the wavepacket. Fig. 5*A* presents an expanded view of normalized wavepackets measured at several separations larger than 10 μm; arrows indicate the time at which the peak amplitude reaches a given distance from the pump. The solid circles in Fig. 5*B* show the displacement of the wavepacket peak as a function of time. Conventionally, the slope of a fit to these points yields the group velocity, *v*_*g*_. From this perspective, the data are quite puzzling, as *v*_*g*_ appears to increase with time, reaching ≈2 ×10^7^ cm/s, a value that is much larger than that expected for spin waves. Finally, Fig. 5*C* presents a zoomed-in view of the wavepacket amplitude vs. separation, now on a double logarithmic plot.

Below we show that the MSW dispersion relation, *ω*(**k**), in biaxial magnets successfully explains the anomalous wavepacket propagation in Fe_3_Sn_2_. Crucially for the interpretation of our experiments, photoexcitation launches a coherent spin wavepacket, comprising a Gaussian distribution of wavevectors that are initially in phase. The time- and position-dependent magnetization detected by TR-MOKE can be calculated using the following relation:

[3] $$\begin{array}{c} \delta M_{z}\left(r,t\right)\propto \;\text{Re}\int z\cdot m\left(k\right)g\left(k\right)e^{ik\cdot r}e^{-i[\omega (k)-i\alpha ]t}d^{3}k, \end{array}$$

with,

[4] $$\begin{array}{c} g\left(k\right)\propto \frac{e^{-\sigma^{2}\left(k_{x}^{2}+k_{y}^{2}\right)/2}}{ik_{z}-1/\delta_{p}}, \end{array}$$

where *g*(**k**) is the Fourier transform of the initial perturbation generated by the pump pulse and **m**(**k**) is the normal mode eigenvector. The radius of the focused laser beam, *σ*, and the anisotropy parameters are determined from independent measurements. The only adjustable parameters in the theory are the damping constant, *α*, and the effective penetration depth, *δ*_*p*_, of the perturbation that induces the subsequent precessional motion. Parameter values, *δ*_*p*_ = 230 nm and *α* = 2.7 × 10^9^ s^−1^, were chosen to achieve the best fit (solid red line) to the amplitude vs. distance data shown in Fig. 5*C*. The same parameters accurately reproduce the anomalous wavepacket position vs. time data as well (red line in Fig. 5*B*), adding additional support for our theoretical model (*SI Appendix*, section V for details).

### Physical Origin of Anomalous Propagation.

In the previous section, we showed that spin wavepacket dynamics in Fe_3_Sn_2_ can be quantitatively modeled by the MSW dispersion relations for a biaxial ferromagnet. In this section, we offer a physical picture that underlies the most puzzling feature of the wavepacket propagation: an apparent velocity that exceeds the expected SW velocity. Essentially, the seemingly anomalous behavior is a consequence of a breakdown of the group velocity description that occurs when a dispersion relation is highly structured within the range of wavevectors that comprise the packet. In the following, we show that dynamics in this regime can lead to early arrival times at remote locations, which we refer to as spin wave precursors.

To illustrate the origin of spin wavepacket precursors, consider the V-shaped dispersion relation for propagation in the *y* direction shown in Fig. 5*D*. In this approximation to the actual relation (Fig. 4*B*), SWs propagate with constant velocity for *k*_*y*_ < 2*k*_*z*_ and do not propagate for *k*_*y*_ > 2*k*_*z*_. The precursor effects arise from SW modes in which *k*_*z*_*σ* is small, such that the Gaussian distribution of photoexcited wavevectors (green line in Fig. 5*D*) spans both propagating and nonpropagating regimes.

The time-evolution of the wavepacket is given by summing the contributions from the two regimes,

[5] $$\begin{array}{cc} & \delta M(y,t)\\ & \;\;\propto 2\cos\omega_{0}t[\int_{0}^{2k_{z}}dk_{y}g\left(k_{y}\right)[\cos k_{y}\left(y-vt\right)+\cos k_{y}\left(y+vt\right)]\\ & \;\;\;+\int_{2k_{z}}^{\infty }dk_{y}g\left(k_{y}\right)\cos\left(k_{y}y\right)], \end{array}$$

where *ω*_0_ is the frequency at *k*_*y*_ = 0 and *v* is the slope of the V-shaped region. Fig. 5*E* shows the propagating and nonpropagating terms in Eq. **5** evaluated at *t* = 0 (red and blue lines, respectively), together with their sum (green line). The individual terms in Eq. **5** are oscillatory with a slowly decaying envelope, as expected for the Fourier transform of a sharply truncated Gaussian. Notice that the oscillations cancel out under summation, yielding the initial Gaussian wavepacket. However, for *t* > 0, the propagating component moves away from the origin at velocity *v*, while the nonpropagating component remains stationary, disrupting the initial cancellation of the two components. This effect manifests as the appearance of oscillations in magnetization at large distances within a short time frame. In this simplified picture, a spin wave precursor can be seen at arbitrarily large distances within a time of order of the precession period. In reality, the range of detection will be limited by the rounding of the dispersion neglected in our V-shape approximation; nevertheless, precursors will appear on time scales that are not set by the SW velocity.

## Conclusion and Outlook

We have shown that spin waves in Fe_3_Sn_2_ can be optically excited, propagated, and detected across large distances (> 20 μm) within short timescales (< 100 ps). The observation of precursors reflects a unique regime of light–matter interaction, resulting from the combination of Gaussian laser excitation and V-shaped magnetostatic spin wave dispersion. We note that this phenomenon is distinct from the Sommerfeld-Brillouin precursor (29) or effects associated with propagation in a regime of strong absorption, as observed for spin propagation on lengths scales ≤50 nm in NiO (30). Here, the potential for applications is the ability to transmit a large bandwidth of spin information across macroscopic distances, on a time scale not limited by the group velocity of the spin waves.

## Materials and Methods

### Crystal Growth.

Single crystals of Fe_3_Sn_2_ were grown using a chemical vapor transport method with conditions outlined in ref. 13. The resulting crystals tend to be hexagonal thin plates, and optical measurements were performed on as-grown (001) surfaces.

### Field Dependence Measurements.

The time-resolved magneto-optic Kerr effect (tr-MOKE) measurements with an out-of-plane magnetic field were performed with 1,560-nm pump and 780-nm probe laser pulses generated from a Menlo C-Fiber erbium fiber oscillator operating at a repetition rate of 100 MHz. The pump and probe powers were set to 20 mW and 0.1 mW and focused onto the sample surface with approximate spot sizes of 20 μm and 6 μm, respectively, using an objective lens with a numerical aperture (N.A.) of 0.25. The transient changes in Kerr rotation values were subsequently measured with a balanced photodetection scheme and a lock-in amplifier. The pump laser pulses were modulated at 100 kHz with a photoelastic modulator (PEM).

### Propagation Measurements.

The nonlocal propagation experiments were carried out with 514-nm pump and 633-nm probe pulses generated from the ORPHEUS-TWINS optical parametric amplifiers pumped by the Light Conversion CARBIDE Yb-KGW laser amplifier operating at the repetition rate of 600 kHz. Both beams were focused onto the sample surface with approximate spot sizes of 6 μm and 5 μm, respectively, with incident laser powers fixed at 30 μW. This power optimized signal-to-noise ratio without generating measurable light-induced average heating. (*SI Appendix*, section VII for further information on the pump power dependence of the wavepacket dynamics.) The position of the pump focus was scanned by adjusting the voltage applied to the 2-axis galvanometer-driven mirrors, which are located at a distance 4*f* (*f* = 50 cm) before the entrance aperture of the final objective lens (N.A. =0.25). A pair of telescope lenses with focal lengths of *f* are placed equidistant from the galvo mirrors and the objective so that the laser beam steered from the galvo mirrors forms a one-to-one image at the entrance of the objective lens. The pump laser pulses were modulated at 100 kHz with a PEM.

## Supplementary Material

Appendix 01 (PDF)

## Data Availability

All study data are included in the article and/or *SI Appendix*.
