# Supplementary material for: Spin wavepackets in the Kagome ferromagnet Fe3Sn2: Propagation and precursors
Source: Proc Natl Acad Sci U S A. 2023 May 15;120(21):e2220589120. doi: 10.1073/pnas.2220589120 (PMC10214209; doi:10.1073/pnas.2220589120)
Supplement: Supplementary file 1 — Appendix 01 (PDF) [file pnas.2220589120.sapp.pdf]

Supporting Information for "Spin wavepackets in the Kagome ferromagnet Fe_3Sn_2: propagation and precursors"

I. SYMMETRY-BASED MICROSCOPIC MODEL

Fe_3Sn_2 has space group $R\bar{3}m$ (#166), in which the magnetic Fe^{x+} ions form a kagome lattice in each layer that are A-B-C stacked in the three-dimensional crystalline structure. Our goal is to describe the spin dynamics of Fe_3Sn_2 by the local moments $\{\vec{S}_r\}$ of Fe^{x+} ions (on lattice site r). Although there are also itinerant electrons present, their contribution to the interactions between Fe^{x+} moments can be incorporated by integrating them out to obtain a pure spin model of local moments $\{\vec{S}_r\}$.

Since Fe_3Sn_2 is in a ferromagnetic ordered phase below $T_c = 657$ K, we expect a ferromagnetic coupling between kagome layers, and therefore focus our analysis on a monolayer model on the two-dimensional kagome lattice. The minimal model compatible with the space group symmetry is the XXZ Hamiltonian:

$$\hat{H}_{XXZ} = -J \sum_{\langle i,j \rangle} S_i^x S_j^x + S_i^y S_j^y + \Delta S_i^z S_j^z, \tag{S1}$$

where Δ is a parameter characterizing the easy-plane anisotropy of the compound. Specifically, when $\Delta < 1$, the ferromagnet favors an in-plane magnetic moment, while $\Delta > 1$ describes an easy-axis ferromagnet whose magnetic moments align along the c-axis. We have neglected the "breathing" terms that distinguish the upper and lower triangle of the kagome lattice, as it is not necessary to explain the observed magnon properties.

Given the spin model, one can use a linear spin wave (LSW) approach to study the magnon dispersion of the system. In the XXZ model, if the ground state is an easy-plane ferromagnet, the magnetic moments can point to any direction in the $a-b$ plane, giving rise to gapless Goldstone modes of the spontaneously broken continuous symmetry $U(1) = \{e^{i\theta \sum_r S_r^z} | 0 \leq \theta < 2\pi\}$. In fact, in the framework of LSW approach, this gapless $\vec{k} = 0$ magnon mode can persist even without a continuous $U(1)$ symmetry of the spin model, summarized in the following statement:

Theorem: *In a bilinear spin model of a spin-orbit coupled magnet, if the long range ferromagnetic order spontaneously breaks the n-fold crystalline rotation symmetry C_n with $n = 3, 4, 6$, there*

will be gapless modes in the magnon spectrum near the zone center $\vec{k} = 0$ using the LSW approach.

Below we explain the reason behind this theorem. Consider a bilinear spin model with C_n rotational symmetry along the c-axis:

$$\hat{H}_2 = \sum_{i,j} \sum_{a,b,=x,y,z} S_i^a R_{i,j}^{a,b} S_j^b, \quad S_{\vec{r}}^+ = S_{\vec{r}}^x + \mathrm{i} S_{\vec{r}}^y \xrightarrow{C_n} e^{2\pi \mathrm{i}/n} S_{C_n \vec{r}}^+. \tag{S2}$$

For ferromagnetic order with ordering wavevector $\vec{Q} = 0$, the spin raising operator S^+ near the zone center Γ transforms as,

$$S_{\vec{k}=0}^+ = \frac{1}{\sqrt{N}} \sum_r S_r^+ \xrightarrow{C_n} e^{2\pi \mathrm{i}/n} S_{\vec{k}=0}^+. \tag{S3}$$

Therefore near the zone center $\vec{k} \approx 0$, any C_n-symmetric bilinear Hamiltonian must have the following form:

$$\hat{H}_2 = \sum_{|\vec{k}| \ll 1} f(\vec{k}) S_{\vec{k}}^+ S_{\vec{k}}^- + h.c. + \cdots \tag{S4}$$

This means that in a generic bilinear spin model, the C_n rotational symmetry for $n = 3, 4, 6$ is enlarged to an emergent $U(1)$ symmetry in the long wavelength ($|\vec{k}| \ll 1$) limit. As a result, spontaneously breaking the C_n symmetry in an in-plane ferromagnetic order will also break the emergent $U(1)$ symmetry near zone center, hence giving rise to gapless magnon modes. These gapless magnons can be thought of as the "pseudo-Goldstone" modes from spontaneously breaking the emergent $U(1)$ symmetry.

The gapless magnon modes, however, are inconsistent with the gap of 8 GHz $\approx$ 0.2 meV that we observe in Fe_3Sn_2. As a result, within the LSW theory, we have to go beyond the bilinear XXZ model described above. Due to Hermiticity and time reversal symmetry, any term in the Hamiltonian must contain an even number of spin operators. To reduce the emergent $U(1)$ symmetry down to C_n, we have to introduce four-spin or six-spin interactions. The lowest order term compatible with the space group $R\bar{3}m$ is a quartic term

$$\hat{H}_4(\vec{k} = 0) \sim f_4(\vec{k} = 0)(S_{\vec{k}=0}^+)^3 S_{\vec{k}=0}^z + h.c., \tag{S5}$$

Unfortunately, for in-plane magnetic order, such a term vanishes in the LSW approximation and doesn't open up a magnon gap at the zone center. As a result, the lowest-order term to explain

the magnon gap at Γ is a six-spin term $\sim (S^+)^6 + h.c.$. In Fe_3Sn_2, the simplest realization of this term is a 3-site ring exchange interaction:

$$\hat{H}_{ring} = -K \sum_{\langle i,j,k \rangle} \left[(S_i^+)^2 (S_j^+)^2 (S_k^+)^2 + h.c. \right], \tag{S6}$$

where $\langle i, j, k \rangle$ denotes nearest neighbor triplets i, j, k on the same triangle.

To summarize, our minimal model to describe the spin dynamics of Fe_3Sn_2 is given as follows:

$$\begin{aligned} \hat{H}_{min} &= \hat{H}_{XXZ} + \hat{H}_{ring} + \hat{H}_{Zeeman} \\ &= -J \textstyle\sum_{\langle i,j \rangle} (S_i^x S_j^x + S_i^y S_j^y + \Delta S_i^z S_j^z) - K \sum_{\langle i,j,k \rangle} \left[(S_i^+)^2 (S_j^+)^2 (S_k^+)^2 + h.c. \right] - B_z \sum_i S_i^z, \end{aligned} \tag{S7}$$

where B_z labels the Zeeman field applied along $c-$axis. At $\vec{k} = 0$, with $B_z = 0$, we can work out the magnon spectrum using a Holstein-Primakoff transformation:

$$S_i^z = S - b_i^\dagger b_i \tag{S8}$$

$$S_i^+ = \sqrt{2S - b_i^\dagger b_i} \cdot b_i \approx \sqrt{2S} \cdot b_i \tag{S9}$$

$$S_i^- = b_i^\dagger \sqrt{2S - b_i^\dagger b_i} \approx b_i^\dagger \sqrt{2S} \tag{S10}$$

where $b_i^\dagger, b_i$ are boson creation and annihilation operators, and S is the total spin on each site i. After making these substitutions, isolating quadratic terms, and converting to momentum space, we can write the boson BdG Hamiltonian as

$$\hat{H}_{LSW} = \sum_{\vec{k}} \hat{\Gamma}^\dagger(\vec{k}) \begin{pmatrix} F(\vec{k}) & G(\vec{k}) \\ G^*(-\vec{k}) & F^*(-\vec{k}) \end{pmatrix} \hat{\Gamma}(\vec{k}) \equiv \sum_{\vec{k}} \hat{\Gamma}^\dagger(\vec{k}) H_b(\vec{k}) \hat{\Gamma}(\vec{k}), \tag{S11}$$

where

$$\hat{\Gamma}^\dagger(\vec{k}) = \left(\hat{b}_{\vec{k}1}^\dagger \ \ldots \ \hat{b}_{\vec{k}m}^\dagger \ \hat{b}_{-\vec{k}1} \ \ldots \ \hat{b}_{-\vec{k}m} \right) \tag{S12}$$

and there are $m = 3$ spins in each unit cell. At $\vec{k} = 0$, we have the simple form

$$F(\vec{k} = 0) = \begin{pmatrix} A & B & B \\ B & A & B \\ B & B & A \end{pmatrix} \tag{S13}$$

$$G(\vec{k} = 0) = \begin{pmatrix} C & D & D \\ D & C & D \\ D & D & C \end{pmatrix}, \tag{S14}$$

where

$$A = 2JS + \frac{3}{2}KS^3(2S-1)^2 \tag{S15}$$

$$B = -JS(1+\frac{\delta}{2}) + 2KS^4(2S-1) \tag{S16}$$

$$C = -\frac{1}{2}KS^3(2S-1)^2 \tag{S17}$$

$$D = -\frac{1}{2}JS\delta - 2KS^4(2S-1), \tag{S18}$$

where we define $\delta \equiv \Delta - 1$. To find the magnon spectrum, we diagonalize the matrix $\sigma_z H_b$, where σ_z acts on the $(\hat{b}, \hat{b}^\dagger)$ space. We find that two of the bands are degenerate at $\vec{k} = 0$

$$E_1 = \sqrt{(A+2B)^2 - (C+2D)^2} \tag{S19}$$

$$E_{2,3} = \sqrt{(A-B)^2 - (C-D)^2}. \tag{S20}$$

For small values $\frac{K'}{J}, |\delta| \ll 1$, the non-degenerate band is lower in energy, with energy given by

$$E_1 = \sqrt{2K'(6S-1)(-2JS\delta + (2S-1)K')} = 2S^2\sqrt{KJ(2S-1)(6S-1)}\Big[1 - \delta + \frac{S^2(2S-1)^2K}{2J}\Big]^{1/2} \tag{S21}$$

where $K' \equiv KS^3(2S-1)$. The experimentally determined spin stiffness of Fe_3Sn_2 is [1]

$$D = 2JSa^2 \approx 231 \text{ meV Å}^2, \tag{S22}$$

where $a = 5.34$ Å is the the lattice constant. Given the gap $E_1 \approx 8$ GHz ≈ 33 μeV, one can estimate the exchange coupling to be

$$K \sim \frac{E_1^2}{JS^6} \sim \frac{E_1^2}{DS^5/2a^2} \sim 0.27 \ \mu\text{eV} \tag{S23}$$

where we have set $S \sim O(1)$ in the estimation. This means a very small and realistic ring exchange coupling can already induce the 0.03 meV magnon gap at the zone center.

II. FREE ENERGY ANALYSIS

Consider a classical ferromagnetic ground state with

$$\langle \vec{S}_r \rangle = (M_x, M_y, M_z), \quad \forall\ r \in \Lambda \tag{S24}$$

the free energy density is given by

$$\mathcal{F} = \frac{\langle \hat{H}_{min} \rangle}{N} = -6J(M_x^2 + M_y^2 + \Delta M_z^2) - 2K\big[(M_x + \mathrm{i}M_y)^6 + h.c.\big] - 3B_z M_z, \tag{S25}$$

where N is the total number of unit cells in the system. Making use the relation,

$$(\vec{S}_r)^2 = S(S+1) = M_x^2 + M_y^2 + M_z^2, \tag{S26}$$

we can expand around the in-plane easy-axis ferromagnetic order $\langle \vec{S}_r \rangle = \sqrt{S(S+1)}(1,0,0)$ and do the LSW expansion,

$$M_x = \sqrt{S(S+1) - M_y^2 - M_z^2} \approx \sqrt{S(S+1)} - \frac{M_y^2 + M_z^2}{2\sqrt{S(S+1)}} + O(\frac{M_{y,z}}{S})^4, \tag{S27}$$

This yields the zero-field free energy density as a function of spin wave fluctuations $M_{y,z} \ll 1$:

$$\mathcal{F}_{LSW}(B_z = 0) = \rho_y M_y^2 + \rho_z M_z^2 + O(M_{y,z}^4), \tag{S28}$$

$$\rho_y = 72[S(S+1)]^2 K, \quad \rho_z = 6J(1-\Delta) + 12[S(S+1)]^2 K, \tag{S29}$$

where S is the total spin of each Fe^{x+} ion. Making use of relation (S26) we can rewrite the above free energy density as

$$\mathcal{F}_{LSW}(B_z = 0) = -\rho_y M_x^2 + (\rho_z - \rho_y) M_z^2 + O(M_{y,z}^2). \tag{S30}$$

III. DIPOLAR MAGNON DISPERSION AND DEPENDENCE ON APPLIED MAGNETIC FIELD

The magneto-crystalline anisotropy can be expressed as the combination of a hard axis along the out-of-plane direction (z-axis) and an easy axis along the in-plane direction (x-axis). The magnetization vector is denoted as $\boldsymbol{M} = (M_x, M_y, M_z)$. The free energy of the anisotropy F_A can be written as

$$F_A = (-K_x M_x^2 + K_z M_z^2)/M_s^2, \tag{S31}$$

where K_x is the in-plane anisotropy energy, K_z is the out-of-plane anisotropy energy (K_x, $K_z > 0$) and M_s is the saturation magnetization. The effective field induced by the anisotropy energy can be derived as

$$\boldsymbol{H}_A = -\nabla_{\boldsymbol{M}} F_A = -\frac{2}{M_s^2}(K_x M_x \hat{x} - K_z M_z \hat{z}). \tag{S32}$$

In addition to $\boldsymbol{H}_A$, the effective field $\boldsymbol{H}_{eff}$ includes the dynamic demagnetizing field $\boldsymbol{h}$, which is the key to consider the magnetic dipole-dipole interaction. Therefore, our goal is to solve the Landau-Lifshitz equation below to calculate the magnon dispersion,

$$
\begin{aligned}
&\frac{d\boldsymbol{M}}{dt} = -\gamma \boldsymbol{M} \times \boldsymbol{H}_{\text{eff}}, \\
&\boldsymbol{H}_{\text{eff}} = \boldsymbol{H}_A + \boldsymbol{h},
\end{aligned}
\tag{S33}
$$

where γ is the gyromagnetic ratio. We assume that the equilibrium magnetization is along the x-direction and consider an infinitely large sample, so that the normal modes have the form of plane waves. We have

$$
\begin{aligned}
&M_x = M_s, \\
&M_y = m_y(\boldsymbol{r}, t) = m_y e^{i(\boldsymbol{k}\cdot\boldsymbol{r}-\omega t)}, \\
&M_z = m_z(\boldsymbol{r}, t) = m_z e^{i(\boldsymbol{k}\cdot\boldsymbol{r}-\omega t)}, \\
&h_x(\boldsymbol{r}, t) = h_x e^{i(\boldsymbol{k}\cdot\boldsymbol{r}-\omega t)}, \\
&h_y(\boldsymbol{r}, t) = h_y e^{i(\boldsymbol{k}\cdot\boldsymbol{r}-\omega t)}, \\
&h_z(\boldsymbol{r}, t) = h_z e^{i(\boldsymbol{k}\cdot\boldsymbol{r}-\omega t)}.
\end{aligned}
\tag{S34}
$$

Since $m_{y,z}$ and $h_{x,y,z}$ are the dynamic magnetization and dynamic magnetic fields, $m_{y,z}$, $h_{x,y,z} << M_s$. Substituting Eq. S34 into Eq. S33, we get the relation between $m_{y,z}$ and $h_{y,z}$.

$$
\begin{bmatrix} m_y \\ m_z \end{bmatrix} = \frac{\gamma M_s}{\omega_T \omega_x - \omega^2} \begin{bmatrix} \omega_T & -i\omega \\ i\omega & \omega_x \end{bmatrix} \begin{bmatrix} h_y \\ h_z \end{bmatrix},
\tag{S35}
$$

where

$$
\omega_x = \frac{2\gamma K_x}{M_s}; \;\omega_T = \frac{2\gamma K_T}{M_s}; \;K_T = K_x + K_z.
\tag{S36}
$$

Noting the relation $\nabla \times \boldsymbol{h} = 0$, we can consider the dynamic magnetic field as the gradient of a scalar potential ψ,

$$
\begin{aligned}
&\psi = a e^{i(\boldsymbol{k}\cdot\boldsymbol{r}-\omega t)}, \\
&\boldsymbol{h} = \nabla\psi = i\boldsymbol{k} a e^{i(\boldsymbol{k}\cdot\boldsymbol{r}-\omega t)}.
\end{aligned}
\tag{S37}
$$

Taking the dipole-dipole interaction into account, we use the Gauss's Law of the Maxwell's equations

$$
\nabla \cdot \boldsymbol{b} = 0,
\tag{S38}
$$

where $\boldsymbol{b} = \boldsymbol{h} + 4\pi\boldsymbol{M}$. Using the relation in Eq. S35, we have

$$\begin{aligned} b_x &= h_x + 4\pi M_s, \\ b_y &= (1 + \frac{\omega_M \omega_T}{\omega_T \omega_x - \omega^2}) h_y - \frac{i\omega_M \omega}{\omega_T \omega_x - \omega^2} h_z, \\ b_z &= \frac{i\omega_M \omega}{\omega_T \omega_x - \omega^2} h_y + (1 + \frac{\omega_M \omega_x}{\omega_T \omega_x - \omega^2}) h_z, \end{aligned} \tag{S39}$$

where $\omega_M = 4\pi\gamma M_s$. Then Eq. S38 becomes

$$\frac{\partial^2 \psi}{\partial x^2} + (1 + \frac{\omega_M \omega_T}{\omega_T \omega_x - \omega^2}) \frac{\partial^2 \psi}{\partial y^2} + (1 + \frac{\omega_M \omega_x}{\omega_T \omega_x - \omega^2}) \frac{\partial^2 \psi}{\partial z^2} = 0. \tag{S40}$$

Recalling that

$$\psi = a e^{i(\boldsymbol{k}\cdot\boldsymbol{r} - \omega t)}, \tag{S41}$$

we have

$$k_x^2 + (1 + \frac{\omega_M \omega_T}{\omega_T \omega_x - \omega^2}) k_y^2 + (1 + \frac{\omega_M \omega_x}{\omega_T \omega_x - \omega^2}) k_z^2 = 0. \tag{S42}$$

By solving Eq. S42, we get the dispersion relation of the dipolar magnon mode,

$$\omega = \frac{\sqrt{\omega_T \omega_x k_x^2 + \omega_T(\omega_M + \omega_x) k_y^2 + \omega_x(\omega_M + \omega_T) k_z^2}}{\sqrt{k_x^2 + k_y^2 + k_z^2}} \tag{S43}$$

The dipolar magnon dispersion relations at selected values of k_z are plotted in Fig. S1. The characteristic frequencies, ω_x, ω_T, and ω_M, are calculated using the parameters obtained from the field dependence of the frequency, as described in the main text. Expressing these parameters in the cgs system used for theoretical calculations, we have, $K_x = 1.76 \times 10^5$ erg/cm^3, $K_z = 2.26 \times 10^6$ erg/cm^3, $M_s = 650$ emu/cm^3, and $\gamma = 1.7 \times 10^7$ Gauss-rad/s. These values yield $\omega_x = 9.2$ GHz, $\omega_T = 127$ GHz, and $\omega_M = 139$ GHz.

We now include an external field applied along the direction of the hard axis (z-axis). The free energy becomes,

$$F = -K_x \frac{M_x^2}{M_s^2} + K_z \frac{M_z^2}{M_s^2} - H_z M_z. \tag{S44}$$

By minimizing the free energy, we show that the equilibrium magnetization in an applied field H_z is $\boldsymbol{M} = (M_s \cos\theta, 0, M_s \sin\theta)$, where

$$\theta = \begin{cases} \arcsin\left(\dfrac{H_z}{H_s}\right), & 0 \leqslant H_z \leqslant H_s \\ \dfrac{\pi}{2}, & H_z > H_s \end{cases} \tag{S45}$$

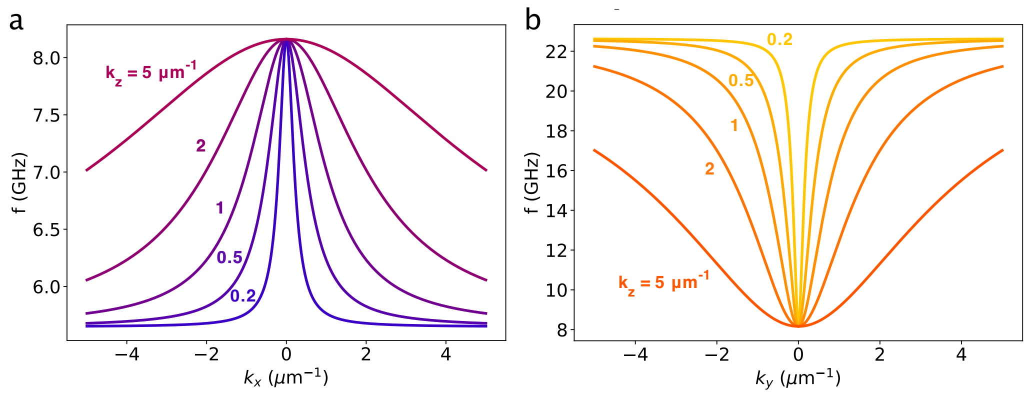

Figure S1. The k_z dependence of the dipolar magnon dispersion. Line cuts taken through the plane defined by (a) $k_y = 0$ and (b) $k_x = 0$ are shown for selected values of k_z.

H_s is the saturation field, and $H_s = 2(K_x + K_z)/M_s$. The normal mode of magnons is a precession in the plane orthogonal to the equilibrium magnetization, so it has the form $\boldsymbol{m} = (-m_a \sin\theta, m_y, m_a \cos\theta)$. Following the similar approach in Sec. III, we have the magnon dispersion below.

For $0 \leqslant H_z \leqslant H_s$,

$$\begin{aligned}
\omega &= \frac{\gamma}{K_x + K_z} \frac{\sqrt{c_x k_x^2 + c_y k_y^2 + c_z k_z^2 - c_{xz} k_x k_z}}{\sqrt{k_x^2 + k_y^2 + k_z^2}}, \\
c_x &= K_x[(H_s^2 - H_z^2)(K_x + K_z) + 2\pi H_z^2 M_s^2], \\
c_y &= (H_s^2 - H_z^2)(K_x + 2\pi M_s^2)(K_x + K_z), \\
c_z &= (H_s^2 - H_z^2) K_x (K_x + K_z + 2\pi M_s^2), \\
c_{xz} &= H_z \sqrt{H_s^2 - H_z^2} K_x M_s^2.
\end{aligned} \tag{S46}$$

For $H_z > H_s$,

$$\begin{aligned}
\omega &= \gamma \frac{\sqrt{d_x k_x^2 + d_y k_y^2 + d_z k_z^2}}{\sqrt{k_x^2 + k_y^2 + k_z^2}}, \\
d_x &= \left(H_z - \frac{2K_z}{M_s}\right)(H_z - H_s + 4\pi M_s), \\
d_y &= (H_z - H_s)\left(H_z - \frac{2K_z}{M_s} + 4\pi M_s\right), \\
d_z &= \left(H_z - \frac{2K_z}{M_s}\right)(H_z - H_s).
\end{aligned} \tag{S47}$$

When $\boldsymbol{k} \to 0$, we get the $k = 0$ frequency as a function of the applied field,

$$\omega(k=0) = \begin{cases} \dfrac{\gamma\sqrt{(H_s^2 - H_z^2)K_x(K_x + K_z + 2\pi M_s^2)}}{K_x + K_z}, & 0 \leqslant H_z \leqslant H_s \\ \gamma\sqrt{\left(H_z - \dfrac{2K_z}{M_s}\right)(H_z - H_s),}, & H_z > H_s \end{cases} \tag{S48}$$

IV. ANGULAR DEPENDENCE OF THE SPIN WAVE PROPAGATION

In this experiment, the transient changes in Kerr rotation were measured as a function of angular orientation between the pump and probe beams, while the spatial separation and time delay between the two pulses are fixed (Fig. S6(a)). The data acquired at various separation distances and time delays between the pump and probe pulses (Fig. S6(b)-(d)) show that the major propagation directions of the spin waves are along $\sim 20°$ and $200°$ with respect to the x axis, in agreement with the bi-directional propagation of forward volume modes.

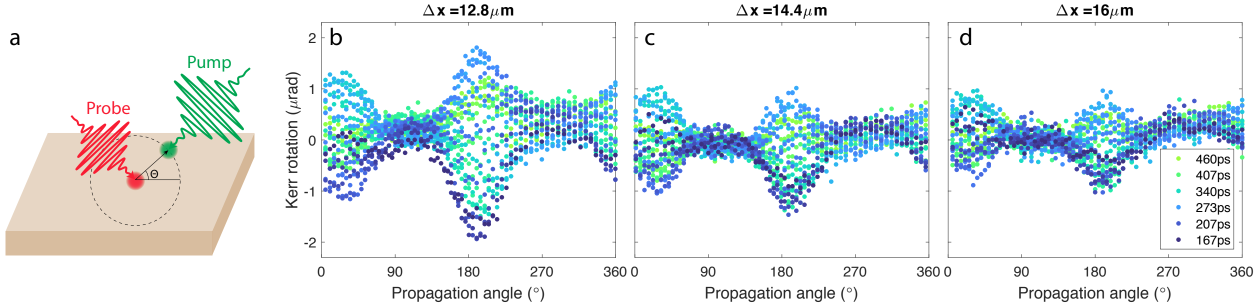

Figure S2. Angular dependence of the spin wave propagation. (a) Overview of the experimental setup where the orientation angle between the pump and probe beams is being continuously varied. (b)-(d) Angle-dependent tr-MOKE measurements taken at pump-probe separation distances of 12.8, 14.4, and 16 μm, respectively. Different colors indicate various time delays between the pump and probe pulses.

The measurements presented in the manuscript were taken within a single domain. Here we present angular dependence measurements taken at three different sample positions labeled in Fig. S3(a). As described in Section IV, the variation in TR-MOKE amplitude was measured as the pump makes a circular scan around the probe at each of the indicated positions. The data plotted in Fig. S3(b)-(d) clearly indicates that the major propagation axes depend on sample position,

indicating the existence of domains.

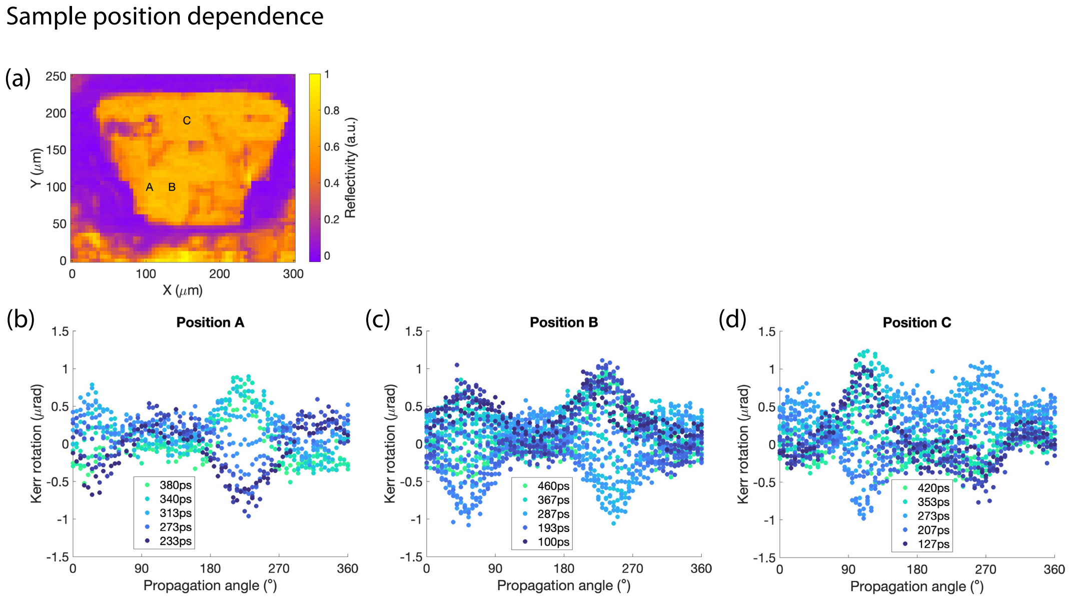

Figure S3. Existence of domains. (a) Reflectivity map of the sample. The letters A, B, and C indicate the three different positions at which the angular dependence measurements were made. (b)-(d) Angle-dependent TR-MOKE measurements at positions A, B, and C, respectively. The pump-probe separation distance was set to 14.4 μm

V. NUMERICAL SIMULATIONS

Numerical simulations are carried out using the following expressions (Eq. (3) of the main text):

$$\delta M_z(\mathbf{r},t) \propto \mathrm{Re} \iiint \mathbf{z}\cdot\mathbf{m}(\mathbf{k})g(\mathbf{k})e^{i\mathbf{k}\cdot\mathbf{r}}e^{-i[\omega(\mathbf{k})-i\alpha]t}dk_x dk_y dk_z, \tag{S49}$$

with

$$g(\mathbf{k}) \propto \frac{e^{-\sigma^2(k_x^2+k_y^2)/2}}{ik_z - 1/\delta_p}, \tag{S50}$$

which describes time and spatial evolution of the magnetization vector after its transient misalignment from the effective anisotropy field direction. The integration bounds were set to $-10/d <$

$k_{x,y} < 10/d$ and $0.01/\delta_p < |k_z| < 3/\delta_p$, where d is the full width at half maximum spot size of the pump beam and δ_p is the effective penetration depth. A Gaussian smoothing was applied to the calculated values of $M_z(r,t)$ to take into account the finite spot size of the probe focus (FWHM $= 5\mu$m). The best fit to the data was found by setting $\delta_p = 230$ nm and $\alpha = 370$ ps, which are the only fitting parameters of the simulations. The time-resolved traces acquired from numerical simulations are in good agreement with the experimental data, as shown in Fig. S4.

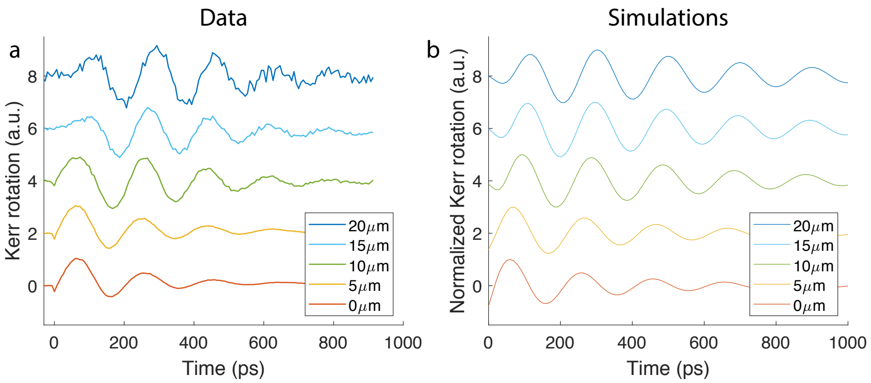

Figure S4. Numerical simulations. (a) Experimental TR-MOKE data traces at selected values of spatial separation Δx between the pump and probe pulses. (b) Numerically simulated TR-MOKE traces chosen at the same values Δx show good agreement with the data

VI. MICROMAGNETIC SIMULATIONS

The micromagnetic simulations are performed using the program MuMax3. The purpose of the micromagnetic simulation is to numerically calculate the magnon dispersion of Fe_3Sn_2 based on a time evolution simulation.

In the simulation, the sample size is set to be 50 μm (length) $\times$ 50 μm (width) $\times$ 3.125 μm (thickness) and the micromagnetic cell size is 0.098 μm $\times$ 0.098 μm $\times$ 0.098 μm. The periodic boundary condition is applied along the x direction. The simulation is performed by setting the saturation magnetization $M_s = 6.495 \times 10^5$ A/m, the exchange constant $A_{ex} = 1.4 \times 10^{-11}$ J/m, the hard-axis anisotropy along z-axis $K_z = 2.317 \times 10^5$ J/m^3 and the easy-axis anisotropy along x-axis $K_x = 1.186 \times 10^4$ J/m^3.

An oscillating external magnetic field along z direction is applied to excite the magnons. To obtain a clear picture of the magnon dispersion, the excitation of the time evolution needs to cover as many wavevectors as possible. Therefore, the spatial profile of the excitation is localized at the very center micromagnetic cell in each xy layer, so that the broadest distribution in k-space can be achieved. The temporal profile of the excitation is a narrow sinc pulse

$$B_z = 0.01\,\mathrm{sinc}[2\pi f(t - t_0)], \tag{S51}$$

where $f = 50$ GHz and the total simulation time $t_0 = 10$ ns. This temporal profile yields a uniform window in the frequency domain to observe the magnon dispersion.

After obtaining the time evolution of each micromagnetic cell, we extract the middle xy layer of the sample and perform a 3D Fourier transform with respect to x, y and t. The Fourier amplitude gives the frequency ω as a function of k_x and k_y, or equivalently the magnon dispersion relation. The simulation results are shown in Fig. S5.

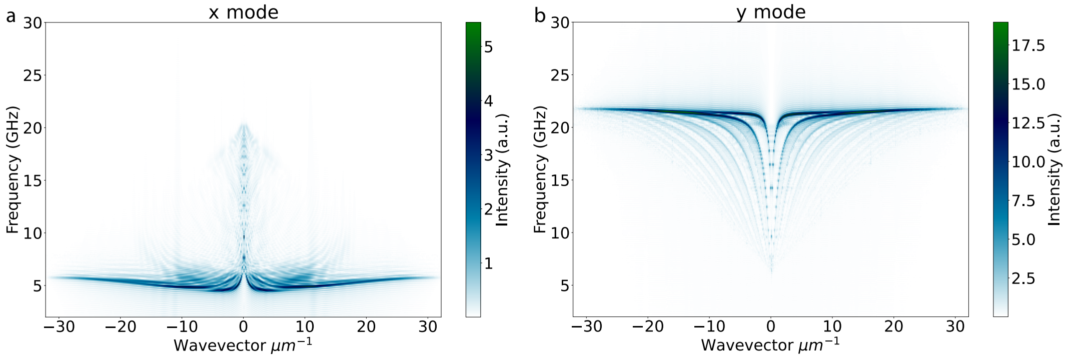

Figure S5. Micromagnetic simulations. The magnon dispersion relations calculated for a biaxial ferromagnet show (a) backward propagating and (b) forward propagating bulk modes, consistent with the calculations described in Section III.

VII. PROPAGATION FOR DIFFERENT PUMP POWER LEVELS

Throughout the measurements presented in the manuscript, the laser power of 30 μW was chosen for both pump and probe pulses as a balance between laser-induced steady-state heating and signal-to-noise. Figure S6 shows measurements of wavepacket propagation for pump laser

powers ranging from 20 to 60 μW, demonstrating that laser-induced heating effects are negligible at 30 μW laser power.

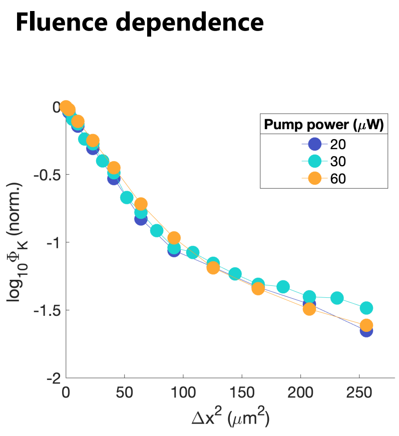

Figure S6. Pump fluence dependence. The log of the amplitude of the Fourier transform of the TR-MOKE data is plotted vs. $(\Delta x)^2$ for different pump laser powers. Laser heating effects are not noticeable in this power range.

[1] R. L. Dally, D. Phelan, N. Bishop, N. J. Ghimire, and J. W. Lynn, Isotropic nature of the metallic kagome ferromagnet Fe_3Sn_2 at high temperatures, Crystals **11**, 307 (2021).
